# Essential facets of competence that enable trust in medical graduates: a ranking study among physician educators in two countries

**DOI:** 10.1007/s40037-013-0090-z

**Published:** 2013-10-19

**Authors:** Marjo Wijnen-Meijer, Marieke van der Schaaf, Kirstin Nillesen, Sigrid Harendza, Olle ten Cate

**Affiliations:** 1 Center for Research and Development of Education, University Medical Center Utrecht, PO Box 85500, 3508 GA Utrecht, the Netherlands; 2 Department of Education, Utrecht University, Utrecht, the Netherlands; 3 Department of Internal Medicine, University Medical Center Hamburg-Eppendorf, Hamburg, Germany

**Keywords:** Competences, Entrustability, Assessment, Supervisors’ perceptions

## Abstract

One way to operationalize the assessment of trainees in a competency-based context is to determine whether they can be entrusted with critical activities. To determine which facets of competence (FOCs) are most informative for such decisions, we performed a Delphi study among Dutch educators. In the current study, the resulting list of facets of competence was evaluated among experienced Dutch and German clinical educators to determine which facets appear most relevant and to evaluate the agreement among experts in different countries as a support for their external validity. Eight Dutch and eight German experts scored each FOC on a five-point scale for relevance. A rank-order comparison showed that there was almost full agreement about the top 10 FOCs, among which ‘Scientific and empirical grounded method of working’, ‘Knowing and maintaining own personal bounds and possibilities’, ‘Active professional development’, ‘Teamwork and collegiality’, ‘Active listening to patients’, and ‘Verbal communication with colleagues and supervisors’. We conclude that these facets of competence may be used in a training for educators who need to make entrustment decisions about trainees.

## Introduction

Following a rapid increase in the popularity of competency-based medical education [1, 2], the methods and concerns around the assessment of competence have been met with increasing interest [3, 4]. Assessment tools in a workplace that cannot be standardized and increased interest in the ‘softer’ skills pose challenges to the assessment procedures [5, 6]. One approach that has been suggested to operationalize the attainment of competencies is to determine whether or when a trainee can be trusted to execute a professional activity without supervision [7]. Trust in trainees requires observations that do not only draw on standardized skills and knowledge but take other facets of competence into account [8, 9]. Using a Delphi approach, we investigated the factors that educators in the Netherlands find important to consider when making entrustment decisions about medical trainees [10]. This yielded a list of 25 relevant factors or ‘facets of competence’ (FOCs) when entrusting trainees with clinical responsibilities.

The aim of the current study was to determine the external validity, i.e. the generalizability, of the factors that Dutch educators found essential. Our approach was to ask experienced educators in two countries to rank-order these FOCs and then to determine the level of agreement among the countries about the highest scoring FOCs.

The study was carried out among Dutch and German medical educators. The Netherlands and Germany differ in medical education culture, particularly in the sense that in the Netherlands education reform has dominated medical curricula throughout the country since the mid-1970s, while such processes in Germany have started only recently [11, 12]. It is fair to say that Dutch medical schools have ‘modern medical curricula’ (not necessarily ‘better’) and most German medical schools have predominantly ‘traditional medical curricula’, while a number of other countries in Europe have positions in between. Agreement among medical educators in the Netherlands and Germany about important FOCs would support the generalizability of those FOCs.

## Methods

### Participants

To find comparable groups in both countries we approached Dutch and German experts. We approached all 24 experienced clinicians in the Netherlands who met the following criteria: (1) holds an academic chair in medical education, (2) works in clinical practice and (3) supervises residents. The 36 German physicians we approached met the following criteria: (1) Master’s degree in medical education, (2) works in clinical practice and (3) supervises residents.

### Questionnaire

The experts were invited by email to complete a questionnaire in an electronic format, after the German version had been translated from the Dutch language. To gain insight into the experts’ judgements about FOCs, they were asked to assign a score from 1 (‘least important’) to 5 (‘most important’) to each of the 25 FOCs that had resulted from the Delphi study. They were requested to give each possible score (1–5) five times (so, 5 FOCs had to get score 1; 5 FOCs had to get score 2, etc.).

### Data analysis

We calculated means, medians and standard deviations for both the Dutch and German group of experts and a level of agreement, according to an adapted De Loe’s [13] procedure. De Loe developed a method to determine the amount of consensus, based on the percentages of answers in one or two contiguous categories of a rating scale (see Table 1). De Loe used this method for a 4-point scale, while we used the same method for a 5-point scale, which makes it a little more stringent.

**Table 1:** Levels of agreement according to De Loe [13]

Agreement	Calculation level of agreement 4-point scale according to De Loe	
	1 category	2 contiguous categories
High (%)	70	80
Medium (%)	60	70
Low (%)	50	60
None	<60 % of ratings in 2 contiguous categories	

## Results

### Respondents

In total 8/24 Dutch and 8/36 German experts participated in the ranking study (response rates 33 and 22 %, respectively). In the Dutch group, 7 were male and the average age was 61 years (56–66 years). In the German group, the average age was 43 years (33–53 years) with 4 male and 4 female responders. The responders in both rounds represented a wide range of surgical and non-surgical disciplines (Dutch: cardiology, general practice, gynaecology, internal medicine, neurology, oncology and surgery; German: emergency medicine, gynaecology, internal medicine, psychiatry and surgery).

## Results

The results of the ranking study are presented in Table 2. The medians and means indicate the ranking of the FOCs. For the Dutch group of responders, medians were between 1 and 5 and the means between 1.63 and 4.75 (SD 0.46–1.64). For the German group, medians varied between 1 and 5 and the means between 1.88 and 4.38 (SD 0.54–1.93). For both groups, the level of agreement varied from none to high.

**Table 2:** Rank-order, median scores, mean scores and level-of-agreement concerning the importance of 25 FOCs for entrustment decisions, as judged by Dutch and German clinical educators

Facets of competence	Rank order				Dutch educators (*N* = 8)				German educators (*N* = 8)			
	Comb.	NL	GE	Ave.Mean	Median	Mean	SD	LoA	Median	Mean	SD	LoA
Scientifically and empirically grounded method of working	1	2	1	4.51	5.0	4.63	0.52	High	5.0	4.38	1.41	High
Knowing and maintaining own personal bounds and possibilities	2	3	3	4.32	5.0	4.63	0.52	High	4.0	4.00	0.93	Low
Active professional development	3	1	5	4.19	5.0	4.75	0.46	High	4.5	3.63	1.69	Low
Teamwork and collegiality	4	7	2	4.01	3.5	3.63	1.06	Low	4.5	4.38	0.74	High
Active listening to patients	5	4	4	3.94	4.0	4.00	0.93	Low	4.0	3.88	1.13	Low
Verbal communication with colleagues and supervisors	6	6	7	3.63	3.5	3.75	1.17	None	3.5	3.50	1.50	None
Empathy and openness	7	10	6	3.44	3.0	3.25	1.04	Low	3.5	3.63	1.30	None
Responsibility	8	5	10	3.38	4.0	3.75	1.39	Medium	3.0	3.00	1.93	None
Coping with mistakes	9	9	9	3.26	3.5	3.38	1.41	None	3.5	3.13	1.64	None
Safety and risk management	10	8	11	3.25	4.0	3.50	1.41	Low	3.0	3.00	1.41	Low
Written (and digital) account/report to colleagues and supervisors	11	11	15	2.94	3.5	3.13	1.13	Medium	3.0	2.75	1.67	None
Attention to individual patient background	12	15	16	2.82	2.5	2.88	1.36	None	3.0	2.75	1.04	Low
Respecting privacy and autonomy of the patient	13	13	17	2.82	2.5	3.00	1.15	None	2.5	2.63	1.30	None
Advising patients	14	14	18	2.75	2.5	3.00	1.20	Low	2.5	2.50	0.54	High
Handling emotions of patients and their relatives	15	12	21	2.69	3.0	3.00	1.20	Low	2.5	2.38	0.74	High
Structure, work planning and priorities	16	20	8	2.65	2.0	2.00	0.93	Medium	4.0	3.29	1.70	None
Ethical awareness	17	17	12	2.63	2.5	2.38	1.30	None	3.0	2.88	1.46	None
Continuity in the care process	18	16	19	2.63	3.0	2.75	1.28	Low	2.0	2.50	1.41	Low
Adapted informing of patients	19	19	13	2.51	2.0	2.13	1.13	Low	3.0	2.88	0.84	Medium
Attention to psychosocial aspects of health problems	20	18	22	2.38	2.0	2.38	1.19	Medium	2.0	2.38	1.19	Low
Active health promotion	21	24	14	2.32	1.5	1.75	0.89	Medium	3.0	2.88	1.25	Low
Financial and social awareness	22	23	20	2.16	1.0	1.88	1.64	High	2.0	2.43	1.51	None
Role differentiation	23	22	23	2.13	1.5	1.88	1.36	High	2.5	2.38	1.30	None
Coping with uncertainty	24	21	24	2.07	1.5	2.00	1.20	Low	1.0	2.13	1.55	Medium
Attention to relatives and caregivers	25	25	25	1.76	2.0	1.63	0.52	High	1.5	1.88	1.36	High

## Discussion

The aim of this study was to determine which FOCs of medical trainees are considered most important to formulate entrustment decisions by experienced supervisors in residency training and to evaluate the agreement about them in different European countries, to expand their generalizability.

We found strong agreement between physician educators from the Netherlands and physician educators from Germany in ranking the competency facets relevant for entrustment decisions. The ‘top 10’ for the total group of responders, based on medians and means, appeared nearly the same as the ‘top 10’ for each of the two countries separately. The only FOC that substantially differed was ‘structure, work planning and priorities’, ranked 8 in the German group, but 20 among the Dutch.

The fact that two groups of physician educators from different medical education cultures highly agreed on the importance of certain FOCs for entrustment decisions strengthens the relevance of these FOCs. The top-10 align with Kennedy et al. [9]’s findings. In their grounded theory study, these authors also found ‘truthfulness’ (the absence of deception) and ‘conscientiousness’ (the thoroughness in data gathering and dependability) to be an important quality that supervisors value in trainees to determine their readiness for independent clinical work [9]. We had not explicitly included these as facets in our list, but it may be assumed that our respondents would score them highly if they had been included. Implicitly, they are reflected in our items that stress scientifically grounded working, openness, responsibility and coping with mistakes, all part of the top-10.

One limitation of our study is the low response, which also varied in the different parts of the study. However, the expertise of the participants and the fact that a wide range of disciplines are included adds to the relevance and the generalized nature of the findings, but the study cannot be viewed as conclusive. Another limitation is that we only included one other country. Future studies to establish a generalized nature of factors that affect entrustment decisions should include countries with other than the Western industrialized culture, as we cannot exclude that those countries would show different factors.

The background of making entrustment decisions deserves further empirical study, preferably with more experts and more countries and focused on a validation in practice.

As a practical outcome, our findings may be used as input for a frame of reference training [14] for clinicians who must regularly take entrustment decisions. Supervisors may be guided by their first impressions of trainees, which may be less accurate than they tend to think [15], and training can make them aware to take multiple facets of competence into account.

## Conclusion

Our aim was to reveal what facets of competence are considered most important for entrustment decisions by supervisors of residents. We found high consensus between experts from the Netherlands and Germany, despite large differences in their curricula. Our findings are relevant for the development of assessment instruments to evaluate whether medical graduates are ready for clinical practice.

## Essentials

- There is high agreement among supervisors about what facets of competence are considered most important for entrustment decisions.
- There is consensus between physician educators from two countries with different medical education climates (the Netherlands and Germany).
